# Therapeutic effect of high-flow nasal cannula on severe COVID-19 patients in a makeshift intensive-care unit: A case report

**DOI:** 10.1097/MD.0000000000020393

**Published:** 2020-05-22

**Authors:** Xiao Lu, Shanxiang Xu

**Affiliations:** Department of Emergency Medicine, Second Affiliated Hospital, Zhejiang University School of Medicine, Hangzhou, China.

**Keywords:** COVID-19, HFNC, makeshift intensive-care unit

## Abstract

**Introduction:**

Several intensive-care units (ICUs) in Wuhan are nonstandard wards that were repurposed from general wards. Considering the shortage of medical resources and the need to prevent nosocomic infection, the respiratory-treatment strategy in these nonstandard ICUs is different from those in general wards and standard ICUs. High-flow nasal cannula (HFNC) plays an important role in nonstandard ICUs and is beneficial to the patients therein.

**Patient concerns:**

In this study, we analyzed four cases of HFNC-treated patients with severe coronavirus disease 2019 (COVID-19) in a makeshift ICU and summarized our experience.

**Diagnoses:**

Four patients diagnosed with COVID-19 according to World Health Organization (WHO) interim guidance were admitted to the makeshift ICU.

**Interventions:**

All patients had oxygen treatment with HFNC, as well as regular treatment of antivirals and traditional Chinese medicine.

**Outcomes:**

Two patients survived after treatment, while the other two died from acute respiratory distress syndrome (ARDS) and heart failure, respectively.

**Conclusion:**

Patients with severe and critical COVID-19 often have poor prognoses after mechanical ventilation, exhibiting corresponding complications such as ventilator-associated pneumonia and deep-vein thrombosis, which significantly prolongs length of stay in the ICU. HFNC could prevent intubation in some patients, thereby avoiding the above complications; however, this needs confirmation in further clinical studies. This treatment reduced difficulty and workloads for healthcare professionals, had good tolerability for patients, might not significantly increase the risk of infection for healthcare professionals, and do not require additional preventive measures against nosocomic infection. HFNC treatment has its advantages in providing oxygen therapy in COVID-19, but healthcare professionals should still pay close attention to changes in patients’ oxygenation rates and respiratory frequency.

## 1 Introduction

In accordance with the national policy of hospitalizing coronavirus disease 2019 (COVID-19) cases to the greatest extent possible, all patients with severe and critical COVID-19 in Wuhan have been admitted to local hospitals. However, because these severe and critical cases have far exceeded the admission capacity of intensive-care units (ICUs) in Wuhan—a problem we think will occur across the world—the National Health Commission of the People's Republic of China has made strategic adjustments, requiring medical-assistant teams from various regions across China to assist Wuhan in establishing some makeshift ICUs during this pandemic. For example, a team from the Second Affiliated Hospital of Zhejiang University School of Medicine (Zhejiang Province, China) has taken over one makeshift ICU in Wuhan to hospitalize and treat severe COVID-19 patients. Because these nonstandard ICUs are repurposed general wards, and considering the shortage of medical resources and the need to prevent nosocomic infection, these wards have different respiratory-treatment strategies than general wards and standard ICUs. High-flow nasal cannula (HFNC) plays an important role and is beneficial to patients in nonstandard ICUs.

HFNC appears to be an effective new therapeutic option compared with other oxygen delivery devices (e.g., non-rebreathing oxygen masks, Venturi masks). It is maximum respiratory flow rate reaches 40 to 60 L/min; it achieves 100% humidification at 37°C and has a positive end-expiratory pressure (PEEP) effect when patients breathe with the mouth closed.^[1,2]^ The major benefits of HFNC include continuous alveolar recruitment and reduction of airway collapse, due to the effect of continuous positive airway pressure (CPAP). Some studies have shown the superiority of HFNC in extubated patients and mild-to-moderate ARDS patients, for whom it reduces length of hospital stay.^[3,4]^

HFNC has a critical role to play in the treatment of COVID-19. The main clinical manifestation of respiratory failure in COVID-19 patients is hypoxemia accompanied by labored breathing.^[5]^ While a noninvasive ventilator has a clear advantage in patients with chronic obstructive pulmonary disease (COPD), noninvasive ventilation (NIV) has low efficiency in reducing labored breathing in severe and critical COVID-19 patients and requires higher inspiratory-pressure support. After withdrawal from NIV, the patient's breathing will rapidly become labored again. However, high inspiratory pressure during NIV causes mask leaks, flatulence, and patient intolerance and can bring about catastrophic complications.^[6]^ PEEP improves oxygenation, but intermittent NIV does not provide continuous PEEP. Respiratory drive in patients with COVID-19 is often increased considerably. Inhaled tidal volume increases during NIV; excessive tidal volume aggravates lung injury.^[7]^ In this study, we analyzed cases of HFNC-treated severe COVID-19 in Wuhan and summarized our experience. Patients or their families gave consent for publication. This report was approved by the Ethics Committee of the Second Affiliated Hospital, Zhejiang University School of Medicine.

## 2 Case presentation

Case #1: A 47-year-old female patient diagnosed with COVID-19, who had been treated for 2 weeks, was transferred from Huoshenshan Mobile Hospital (Wuhan) due to her worsening condition. On admission, she was given oxygen via an oxygen mask (10 L/min; oxygen saturation [SpO2], 92%), which was replaced by HFNC (initial flow rate, 50 L/min; oxygen concentration, 80%; temperature, 37°C). This patient had doubts about and initially refused HFNC. However, after full communication and explanation, she agreed to switch to it. Under HFNC, she gradually relaxed, her respiratory rate gradually slowed down, and her SpO2 rose to 93% to 95%. This patient felt good about her condition and tolerated prolonged use of HFNC for 7 days. After 12 days’ treatment; she recovered from COVID-19 and was transferred to the general ward.

Case #2: A 64-year-old male patient was diagnosed with COVID-19 and dilated cardiomyopathy. While waiting for a heart transplant, he contracted COVID-19. He was given oxygen (10 L/min) on admission due to nasal congestion (SpO2, 88%). This patient and his family members refused treatment with a noninvasive ventilator and mechanical ventilation due to the potential of severe heart failure in dilated cardiomyopathy. Due to his obvious chest tightness, shortness of breath, and orthopnea—all of which might have been partial manifestations of heart failure—he was given HFNC (initial flow rate, 60 L/min; oxygen concentration, 90%; temperature, 37°C) and diuretic treatments. His SpO2 increased to ∼93% to 95%, and his respiratory rate was also reduced. He felt that his chest tightness and shortness of breath were partially relieved. However, he could still not lie flat. This patient refused invasive ventilation treatment and eventually died of severe heart failure and respiratory failure after 4 days of treatment with HFNC.

Case #3: A 57-year-old female patient diagnosed with COVID-19 was experiencing chest tightness and shortness of breath. She was given mechanical NIV on admission with inspiratory positive airway pressure of 18 cm H_2_O, expiratory positive airway pressure of 8 cm H_2_O, respiratory rate of 35 breaths per min (bpm), tidal volume of 350 to 550 mL, and SpO2 of 92% to 94%. This patient was extremely intolerant of NIV and refused to continue it, switching instead to HFNC treatment (initial flow rate, 60 L/min; oxygen concentration, 90%; temperature, 37°C). Her SpO2 increased to ∼93% to 95%, and her respiratory rate was also reduced. She felt that her symptoms of chest tightness and shortness of breath were significantly alleviated, and she could tolerate HFNC treatment. The patient was transferred to nasal-cannula oxygen therapy (3 L/min) after 5 days’ HFNC treatment and was discharged from the hospital after 10 days.

Case #4: A 72-year-old male patient diagnosed with COVID-19 had a history of hypertension and percutaneous coronary-intervention (PCI) surgery. He was given oxygen on admission through an oxygen mask (10 L/min; SpO2, 85%). The mask was then replaced with HFNC (initial flow rate, 60 L/min; oxygen concentration, 90%; temperature, 37°C). His SpO2 increased to ∼91% to 93%, and his respiratory rate was also reduced. This patient felt that his symptoms of chest tightness, shortness of breath, and discomfort were significantly improved after HFNC treatment. However, his condition worsened 2 days later, with decreased SpO2. Intubation was performed successfully; settings for mechanical ventilation were 80% fraction of inspired oxygen (FiO2), 33 cm H_2_O peak inspiratory pressure (PIP; 1 cm H_2_O = 0.098 kPa), and 14 cm H_2_O PEEP. His oxygenation index improved significantly. However, this patient died of severe ARDS after 6 days’ treatment in the ICU.

## 3 Discussion and conclusion

### 3.1 Therapeutic effect

Patients with COVID-19, especially severe and critical cases, generally require oxygen therapy for more than 2 weeks, even up to 1 month. Oxygen therapy is the most basic and critical treatment for these patients. The most important clinical manifestations of respiratory failure in patients with COVID-19 are hypoxemia and labored breathing. A previous study has confirmed that application of HFNC in patients with mild to moderate ARDS reduces the intubation rate,^[8]^ which also meets the clinical needs of patients with COVID-19. One retrospective cohort study^[9]^ showed that the 41 of the 191 patients in the cohort used HFNC for oxygen treatment, and only 26 patients were treated with NIV. As our experience in Wuhan showed, HFNC's effectiveness has also been proven in COVID-19 by improvement in SpO2; however, there was no evidence it could reduce mortality. Patients with severe and critical COVID-19 often have poor prognoses after mechanical ventilation and exhibit corresponding complications, such as ventilator-associated pneumonia and deep-vein thrombosis, which significantly prolongs length of ICU stay. HFNC can prevent intubation in a few patients, thereby avoiding the above complications; however, this needs confirmation in further clinical studies. We set the initial flow rate of HFNC at 40 L/min, and it could be increased to a maximum of 60 L/min. FiO2 was adjusted to maintain SpO2 as indicated by a pulse oximetry reading of 93% to 95%.

### 3.2 Reduced difficulty and workload for healthcare professionals

Considering that the nonstandard ICUs mentioned in this study are repurposed general wards, patients are mostly scattered into single or double rooms, which increases difficulty in providing nursing care. The use of noninvasive ventilators requires adjustments of ventilator parameters and monitoring of patients’ tolerance, which can further increase workload and difficulty for nurses and on-duty physicians. By comparison, HFNC is easier to use. A central monitor that assesses changes in HFNC patients’ oxygenation and respiratory rates also reduces the workloads of nurses when patients have high tolerance and are highly cooperative.

### 3.3 Good tolerability for patients

Most patients tolerate HFNC well, especially when they need long-term oxygen therapy, which greatly minimizes lack of cooperativity or refusal of treatment due to intolerance. However, before applying HFNC, it is still necessary to explain the details to patients, because a small number of them do not tolerate high-flow airflow or experience fear during treatment. Therefore, patient, careful explanation before applying HFNC is necessary to relieve patients’ anxiety, let them actively cooperate with the treatment, and build their confidence that it will cure their disease. Because there is no need to remove the oxygen mask while eating, HFNC treatment does not interfere with eating and does not cause SpO2 reduction, thereby avoiding the adverse consequences of inability to eat. Some patients receiving HFNC treatment choose enteral nutrition, which avoids the high risk of infection associated with indwelling nasogastric tubes and also reduces reflux inspiration due to gastrointestinal flatulence caused by noninvasive ventilators.^[10]^

### 3.4 Prevention of nosocomic infection

As nonstandard or makeshift ICUs are not equipped with negative-pressure rooms, only with general exhaust systems, the virus concentration therein is higher than in standard ICUs, making nosocomic infection prevention urgent. Since air leakage is common in NIV and the exhalation valve is usually located near the patient, it is necessary to consider that NIV could increase the risk of viral spread in exhalations.^[11]^ Studies have shown that as the flow rate setting of HFNC is increased from 10 to 60 L/min, the exhaled-air diffusion range increases from 65 ± 15 mm to 172 ± 33 mm. HFNC has a limited range of exhaled-gas diffusion and does not cause the virus to spread when the respiratory interface is properly worn.^[12]^ Another study also showed no significant difference in Gram-negative *Bacillus* count or in particle sedimentation sampling of 0.4 or 1.5 m under treatment per hour in air specimens between an HFNC group and an oxygen mask group. This indicates that use of HFNC is not associated with increased air or contact surface contamination of Gram-negative *Bacillus* or other bacteria, suggesting that no additional infection control measures are needed.^[13]^ The abovementioned two studies suggest that HFNC might not significantly increase the risk of infection for healthcare professionals and does not require additional preventive measures to be taken against nosocomic infection. We believe that application of HFNC is therefore suitable for temporary or makeshift ICUs. Patients should also wear surgical masks if their conditions allow, so as reducing aerosol transmission.

### 3.5 Other precautions

Although HFNC treatment has its advantages in oxygen therapy for COVID-19, healthcare professionals should still pay close attention to changes in patients’ oxygenation rates and respiratory frequency. If the patient's condition continues to deteriorate, further mechanical ventilation should be considered to avoid delaying intubation, which results in increased mortality. Intubation should be performed when the patient has persistent or progressive signs of respiratory failure and meets at least two of the following criteria after technical problems caused by equipment failure are ruled out^[14]^:

1. respiratory rate >40 bpm;
2. no sign of improvement in high respiratory load;
3. large quantity of airway secretions;
4. respiratory acidosis (pH < 7.35); or
5. <90% SpO_2_ for at least 5 min.

## Author contributions

All authors were major contributors in the writing of the manuscript. All authors approved the final manuscript.
